# Development of Mobile Robot-Based Precision 3D Position Measurement System

**DOI:** 10.3390/s25113261

**Published:** 2025-05-22

**Authors:** Pilgong Choi, Jeng-O Kim, Myeongjun Kim, Kyunghan Kim

**Affiliations:** 1 Department of Laser & Electron Beam Technologies, Advanced Manufacturing Systems Research Division, Korea Institute of Machinery & Materials, Deajeon 34103, Republic of Korea; pgchoi@kimm.re.kr (P.C.); jokim@kimm.re.kr (J.-O.K.); myeongjun@kimm.re.kr (M.K.); 2 Department of Mechanical Engineering, Chungnam National University, Deajeon 34134, Republic of Korea

**Keywords:** dry-dock, 3D laser tracker, 3D point cloud, accuracy of measurement

## Abstract

This study presents an automated docking block placement system developed for regular and emergency repairs of large ships and naval vessels. Traditional methods involve manually arranging heavy concrete docking blocks using cranes or forklifts, which can take several days and pose significant safety risks because of the heavy materials involved. The proposed system integrates an unmanned crane with a six-degree-of-freedom (6-DOF) robotic platform and a mobile robot-based 3D precision positioning system to automate block relocation. The use of a 3D laser tracker mounted on the mobile robot is the key to the system, which, when combined with environmental sensors such as LiDAR and RTK-GPS, provides millimeter-level positional feedback. To address the lack of clear reference points in conventional docking blocks, a precisely machined aluminum target block was attached to each block. An algorithm employing Density-Based Spatial Clustering of Applications with Noise (DBSCAN), KD-Tree, and Random Sample Consensus (RANSAC) techniques was used to detect and classify the vertex of the target block from the 3D point cloud data. The experimental results demonstrated a positional measurement error within 0.5 mm at an 8 m distance. This novel system reduces the setup time, enhances worker safety, and increases the overall efficiency and capacity of dry dock maintenance operations.

## 1. Introduction

### 1.1. Research Background

Dry docks are used for regular and emergency maintenance of ships and naval vessels. Docking blocks are commonly used to position ships in dry docks, and their placement varies depending on the displacement of the vessel and hull shape [1]. In shipyards that repair vessels of various sizes and purposes, the docking blocks must be repositioned for each new vessel, which requires repeated adjustments. Over the past several decades, automated systems have been developed and implemented to reduce the preparation time for docking ships and naval vessels during dry docking. Syncrolift in Vestby, Norway, is a leading global company that provides automated docking systems for the rapid maintenance of small vessels and naval ships [2]. One notable technology for the dry docking of small vessels is the use of bilge support and side support arms, which operate based on a hydraulic system instead of the traditional docking blocks. This system has significantly reduced the preparation time for small-vessel repairs, ensured worker safety, and improved economic efficiency.

However, automated docking systems for reducing the maintenance time of large ships and naval vessels have not yet been developed. Automated systems used in small vessels have limitations in structural stability when applied to large ships and naval vessels. The process of arranging docking blocks requires repositioning dozens of blocks according to the shape of the ship, which takes several days to complete. Consequently, ships requiring urgent repair may have to wait at sea for several days. Furthermore, with the continuous increase in the number of large ships and naval vessels, the waiting time for maintenance is expected to increase. Even today, large ships and naval vessels are positioned in dry docks using traditional concrete blocks that are moved and arranged using cranes or forklifts. To align these blocks with the dimensions specified in the docking plan, multiple workers must manually fine-tune their positions within a margin of a few millimeters while working in close proximity. The use of concrete docking blocks and a manual relocation process involving cranes present ongoing safety hazards.

To address these issues, an automated docking block positioning system for regular and emergency maintenance of large ships and naval vessels has been proposed. This system can ensure worker safety and reduce the preparation time for maintenance, which is expected to enhance the annual maintenance efficiency of dry docks.

### 1.2. Dry Dock Automation System and Research Objectives

The use of the existing manned cranes and concrete blocks is recommended for the proposed dry dock automation system, and its development is currently underway.

**Figure 1:**
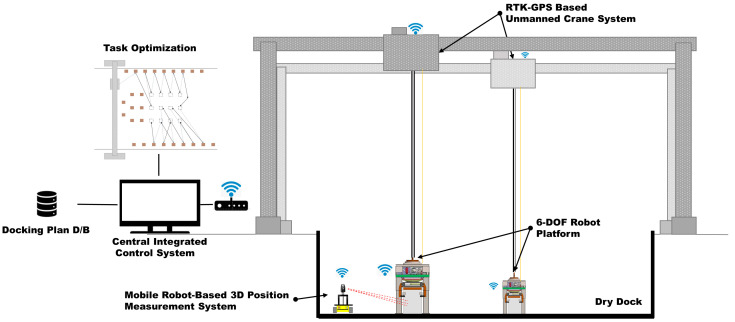
Configuration of the dry-dock automation system.

The configuration of the proposed system is illustrated in Figure 1. Existing manned cranes have been improved to facilitate control based on unmanned crane systems. To automate concrete block transportation, a 6-degree-of-freedom (6-DOF) robot platform was integrated with an unmanned crane. The 6-DOF robot platform fastens and releases the blocks to transport them using an unmanned crane. Furthermore, the positional accuracy of the unmanned crane-based system is expected to achieve an error margin in the order of tens of millimeters during block placement. To minimize such placement errors, a structure capable of adjusting errors of approximately 150 mm without crane assistance was proposed, which enables concrete block placement with an error margin of a few millimeters.

In addition, a measurement system combining a 3D laser tracker and mobile robot was proposed. The positional accuracy of RTK-GPS is in the order of tens of millimeters, typically exhibiting a positional error of approximately 20 mm [3,4,5]. To achieve the target placement error of commercial blocks within a few millimeters, both the control precision of the 6-DOF robot and the measurement precision of the system that provides feedback on the placement position must be excellent. To this end, a mobile measurement system that integrates a 3D laser tracker sensor with a mobile robot is proposed. As the area of the dry dock where the blocks are placed exceeds the maximum measurement radius of 80 m of the 3D laser tracker, a high-precision measurement system capable of extending the measurement radius was required.

The point cloud data measured by the 3D laser tracker contained X-, Y-, and Z-coordinate data relative to the sensor. From this dataset, it was necessary to define a reference point representing the position of a commercial block and develop an algorithm to automatically extract it.

A system integrating a 3D laser tracker and mobile robot was designed and implemented in this study and an algorithm was developed to automatically extract the reference points from the data measured by the 3D laser tracker. To analyze the measurement precision, precisely machined blocks were measured and the 3D data extracted using the developed algorithm were compared with the design dimensional data of the blocks.

## 2. Design of Mobile Robot-Based Precision 3D Measurement System

The objective of this study is to develop an automated system capable of placing dry-dock blocks in a dry-dock environment with a positioning accuracy of a few millimeters. To achieve this, the block transport system must be precise and the measurement system that detects the block position and provides feedback on the absolute positioning errors must also possess high accuracy. Representative outdoor positioning systems include GPS, LiDAR, and ultra-wideband (UWB); however, placing blocks with submillimeter precision requires sensors with submillimeter measurement resolution [6,7,8]. Therefore, a measurement system based on a 3D laser tracker was adopted in this study. The 3D laser tracker supported both reflector-based measurements and surface scanning. The model used in this study was an ATS600 by Hexagon, Stockholm, Sweden. According to the specifications of the manufacturer, the direct scanning mode has a measurement accuracy of ±300 μm at a distance of 60 m. In reflector-based measurements, it can measure up to 80 m with a positioning error of approximately ±500 μm [9,10,11,12]. This system provides block position feedback with millimeter-level accuracy, thereby achieving the desired precision in block placement.

However, the maximum measurement range of a 3D laser tracker is limited to 80 m, whereas the dry dock used in this study spans an area of 250 m × 50 m. Therefore, a single 3D tracker cannot cover the entire workspace, and multiple sensors are required to ensure full coverage. Instead of deploying multiple fixed trackers, this study proposes a system that integrates a single 3D laser tracker with a mobile robot. The proposed system was designed and implemented accordingly. Although fixed trackers offer stable operation, the measurement accuracy decreases with distance. Alternately, mobile platforms enable the sensor to approach the target, thereby enhancing accuracy. Furthermore, the use of multiple sensors may result in idle sensors depending on the block placement location. To overcome these limitations and maximize the advantages of the 3D laser tracker, a mobile-robot-integrated measurement system is proposed. To fully automate the block positioning process, the mobile robot navigates to the designated measurement locations, whereas the 3D laser tracker performs automated measurement and data analysis.

**Figure 2:**
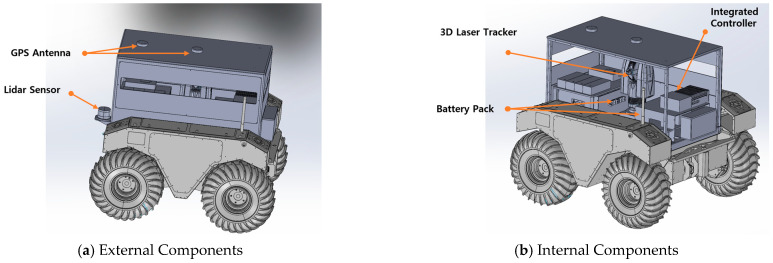
A 3D modeling approach that fuses a mobile LiDAR system with a 3D laser tracker. (**a**) External sensor configuration of the robot. (**b**) Internal sensor and device configuration.

Figure 2 shows the 3D model of the proposed system. The mobile robot is based on the Warthog platform of Clearpath Robotics and is equipped with RTK-GPS and LiDAR sensors for outdoor autonomous navigation. The RTK-GPS provides feedback on the position and heading of the robot with an accuracy of several centimeters, which enables computation of the relative distance and direction to the target position for autonomous path planning [13,14,15,16,17]. The LiDAR sensor maps the dry dock environment in 3D and detects obstacles, thereby enabling the generation of an optimal path from the current location to the measurement point based on the constructed 2D map.

**Figure 3:**
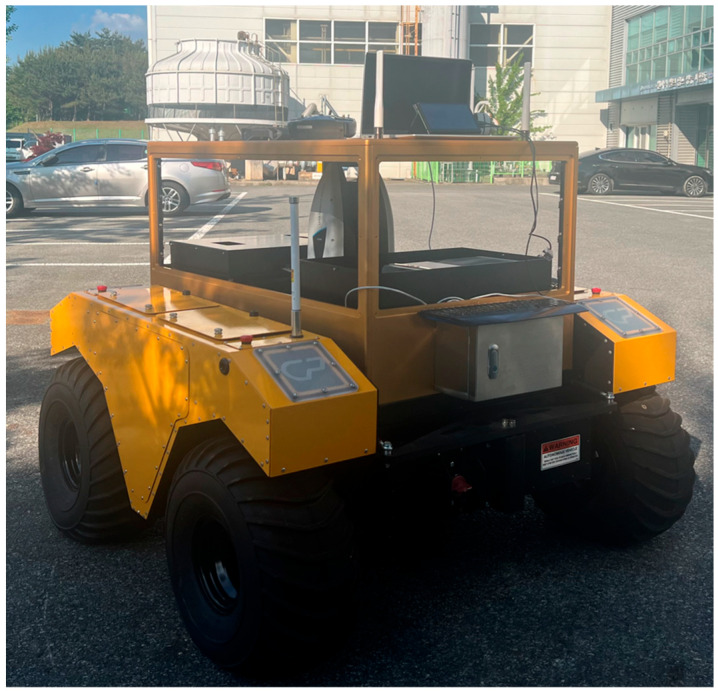
Mobile robot-based 3D position measurement system.

A 3D laser tracker was installed at the center of the robot, along with an integrated controller and battery system. Figure 3 shows an outdoor test of the developed mobile robot-based 3D measurement system.

## 3. Docking Block Position Extraction Algorithm

### 3.1. Environmental Setup

The dry-dock blocks were constructed using concrete and reinforcing steel and, therefore, their surfaces contain concrete. Wooden supports were attached to the top surface to prevent damage to the hulls of ships and vessels during docking. To measure the central position of each support block, it is necessary to measure the positions of its corners accurately. Once the positions of one corner and directions of the two adjacent sides connected to that corner are known, the central position of the block and rotation angle relative to the reference axes of the dry dock can be determined using the design specifications of the block.

However, because the blocks are made of concrete, even with a high-precision measurement system, the measurement accuracy can degrade owing to surface roughness and inherent manufacturing tolerances. This inconsistency makes it challenging to obtain reliable feedback from measured positions, potentially causing variations in the performance of the developed system.

**Figure 4:**
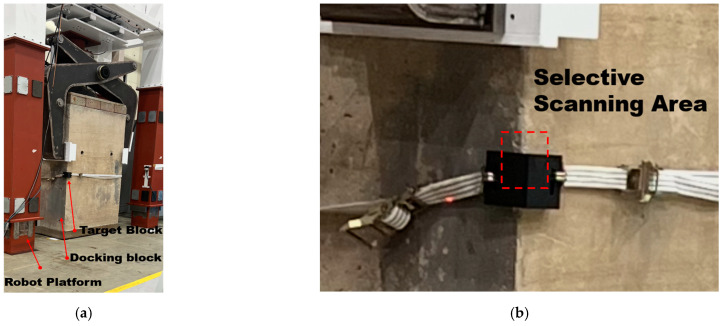
Installation of a processed target block on the dry-dock block. (**a**) The target block setup. (**b**) The 3D laser tracker selective scanning area.

To address this issue, precisely machined aluminum target blocks were fabricated separately and attached to the corners of the dry-dock blocks. Figure 4 shows the aluminum target blocks mounted at the corners of the dry-dock blocks positioned within the developed robotic platform. The target blocks were securely fastened to the support blocks using belt-type fixing straps. The precisely machined upper and lower edges of these aluminum target blocks were designated as representative reference positions for the dry-dock blocks.

### 3.2. Reference Point Extraction Algorithm

To measure the three-dimensional positions of the vertices of the target blocks installed on the docking blocks, the proposed method uses a 3D laser tracker to scan the target area and collect point cloud data. The collected point cloud data is represented as a set X, which consists of N discrete points in three-dimensional space. Each point $P_{i}$ is defined by its Cartesian coordinates $\left(x_{i},y_{i},z_{i}\right)$, as shown in Equation (1):

(1) $$X=\left\{P_{i}=\left(x_{i},y_{i},z_{i}\right)\right|i=1,2,\cdots ,N\}$$

**Figure 5:**
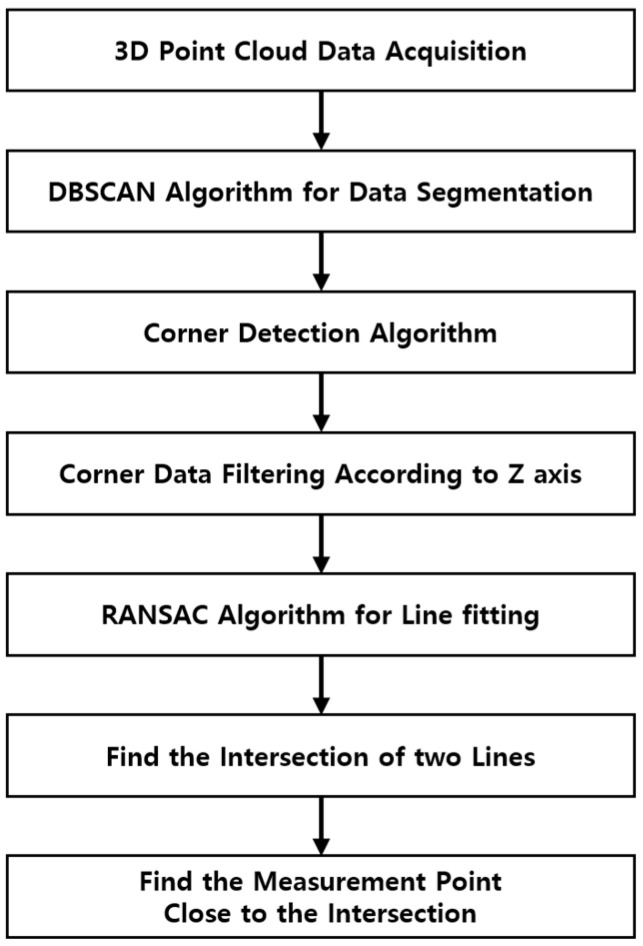
Point extraction algorithm.

The core functionality of the proposed dry-dock block placement automation system involves automatically positioning the blocks, measuring their locations, and providing positional feedback. Therefore, it is essential to develop an algorithm that can automatically analyze point cloud data obtained from a 3D measurement system and extract the reference positions of the target blocks. The algorithm developed to identify and extract the corners of the target blocks from the collected point data is shown in Figure 5.

**Figure 6:**
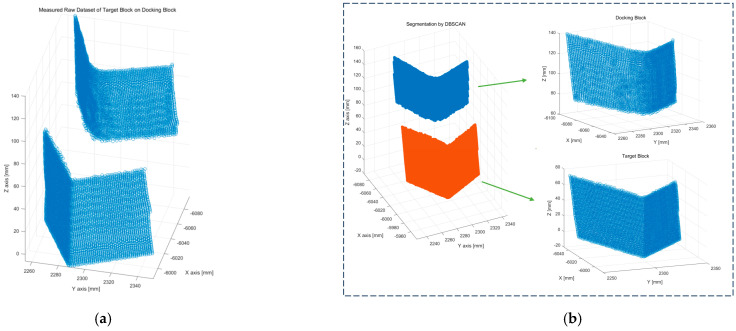
The 3D point cloud raw data segmentation by DBSCAN. (**a**) Point cloud raw dataset. (**b**) Segmentation by DBSAN.

First, it is necessary to separate the target blocks from the concrete blocks (dry-dock blocks) within the collected 3D point cloud dataset. Figure 6 shows the results of clustering the 3D point cloud data acquired by scanning the region of interest containing the target blocks attached to dry-dock blocks using the density-based spatial clustering of applications with noise (DBSCAN) algorithm [18,19,20]. 

The DBSCAN algorithm was developed specifically to cluster spatial data and eliminate noise. It classifies data points by analyzing the relationships between ε-neighborhood points within a predefined distance parameter. After separation from the raw dataset, each dataset, representing the target and concrete blocks, undergoes a vertex extraction process and subsequent detection of two connected straight edges. For every point, a between ε-neighborhood is computed. A point $p_{i}$ is classified as a core point if the number of points within its ε-neighborhood, as shown Equation (2):

(2) $$N_{\varepsilon }\left(p_{i}\right)=\{p_{j}\in P||p_{j}-p_{i}{|}_{2}\leq \varepsilon \}$$

(3) $$\left|N_{\varepsilon }\left(p_{i}\right)\right|\geq Min.P$$

**Figure 7:**
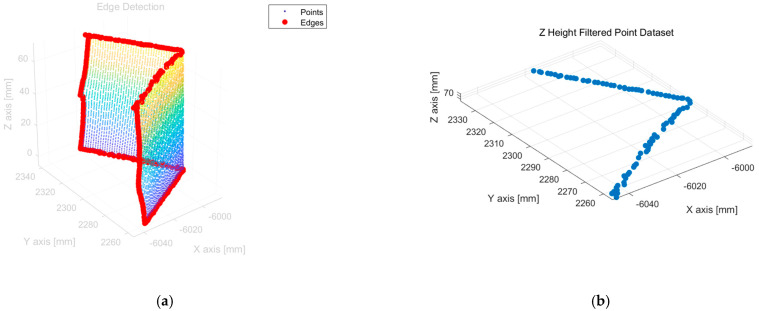
The process of extracting corner (edge) data features after steps (**a**,**b**). (**a**) Edge data filtering. (**b**) Filtered data by Z-axis threshold.

The reference corner points within the separated 3D point cloud dataset of the target block object were included in the subset representing the edges. Therefore, an algorithm was initially executed to classify the edge data. Figure 7a shows the identified edge data, which are highlighted in red. To extract edge data exclusively, a k-dimensional (k-D) tree was first applied to segment the point cloud into neighboring point subsets around each point [21,22,23]. Subsequently, the centroid of the k-nearest neighboring points within each subset was computed. By analyzing the distance between each point and the calculated centroid, the points exceeding a predetermined distance threshold were classified as edge data.

The corner points of the target block comprise two points located at either the upper or lower intersections, where the two surfaces of the target block meet. To extract the positions of the upper corner points, the classified edge dataset is filtered based on the height values using a specified threshold. Figure 7b illustrates the filtered edge data obtained by applying the height-based filtering criterion.

To classify edge points, the k nearest neighbors of each point $p_{i}$ are first identified using a k-dimensional (k-D) tree, as given by

(4) $$N_{k}\left(p_{i}\right)=\mathrm{kNN}\left(p_{i}\right)$$

The local centroid $c_{i}$ of these neighbors is then calculated as the arithmetic mean:

(5) $$c_{i}=\frac{1}{k}\sum_{p_{j}\in N_{k}\left(p_{i}\right)}p_{j}$$

This centroid represents the local geometric center, which is later used to identify points deviating from the neighborhood as edge candidates. Subsequently, the distance between each point and its local centroid was calculated, and points exceeding the threshold $\tau_{d}$ were classified as edge points:

(6) $$\mathrm{classified}\;\mathrm{as}\;\mathrm{edge}\;\mathrm{if}\;d_{i}\geq \tau_{d}$$

Finally, to extract only the upper or lower edge lines of the block, the classified edge points were filtered by their height (*z*-axis) using a predefined threshold $\tau_{z}$:

(7) $$p_{i}\in P_{\mathrm{edge}},\;\mathrm{keep}\;\mathrm{if}\left|z_{i}-z_{\mathrm{ref}}\right|\leq \tau_{z}$$

In the previous step, the classified edge dataset was projected onto a two-dimensional plane by removing the height coordinate (*z*-axis) information. The corner point-of-interest on the target block can be represented as the intersection of two line segments. Therefore, to identify these two line segments from the projected 2D data, a random sample consensus (RANSAC) algorithm was applied. The RANSAC algorithm is widely used for robust line fitting in datasets that may include outliers [24,25,26]. The set of observed data points is defined as follows:

(8) $$D_{obs.}=\{\left(x_{i},y_{i}\right)|i=1,\ldots ,N\}$$

In each iteration t, a minimal subset of points is randomly selected to estimate the model:

(9) $$S_{t}=\{\left(x_{s1},y_{s1}\right),\left(x_{s2},y_{s2}\right)\},\left|S_{t}\right|=5$$

where s = 5 corresponds to the minimum number of points required to define a line.

The model parameters (line coefficients) are estimated from the selected points:

(10) $$a_{t}=\frac{y_{s2}-y_{s1}}{x_{s2}-x_{s1}},b_{t}=y_{s1}-a_{t}x_{s1}$$

For each data point, the residual is computed, and the inlier set $I_{t}$ is defined by comparing the residual to the predefined threshold $\tau_{r}$:

(11) $$r_{i}^{\left(t\right)}=\left|y_{i}-\left(a_{t}x_{i}+b_{t}\right)\right|,I_{t}=\{i|r_{i}^{\left(t\right)}\leq \tau_{r}\}$$

The quality of the model is evaluated by the number of inliers:

(12) $$\mathrm{score}\left(t\right)=\left|I_{t}\right|$$

The model with the highest number of inliers is selected as the best model:

(13) $$\left(a^{*},b^{*}\right)=arg\;max_{\left(a_{t},b_{t}\right)}\;\mathrm{score}\left(t\right),\;\;\;\;\;\;\;\;\;I^{*}=I_{t^{*}}$$

The required number of iterations k to achieve a desired confidence level p is estimated as $k\geq \frac{\ln\left(1-p\right)}{\ln\left(1-w^{s}\right)}$, where ω is the estimated inlier ratio, and s = 5 for the line model.

**Figure 8:**
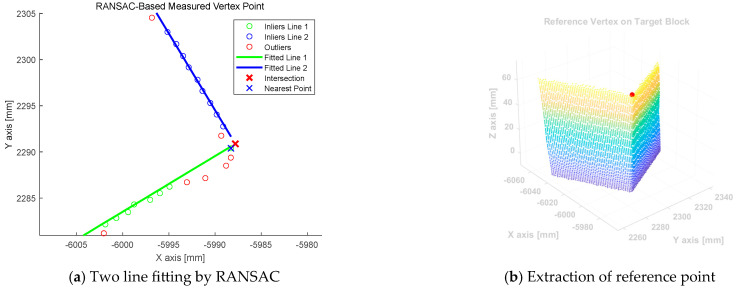
(**a**) Process of fitting the two lines and determining the point closest to their intersections. (**b**) Extracted reference points in three-dimensional space.

Using this approach, two line segments were identified and their intersection points were determined. The angle between the two segments was calculated using the inner product, which was used as a reference to distinguish between the target and the concrete blocks. Figure 8a shows the two line segments fitted using RANSAC and the calculated intersection points. Because the intersection point is a computed point in the 2D plane instead of an actual measured point, the algorithm searches for the nearest measured data point to this intersection within the 2D projected dataset. Then, the height information (*z*-axis) of the nearest point was restored and the final reference point was reconstructed in 3D space. Figure 8b shows the position of the reference point identified using the proposed algorithm.

## 4. Algorithm Verification and Accuracy Evaluation

### 4.1. Design of the Verification Specimen

**Figure 9:**
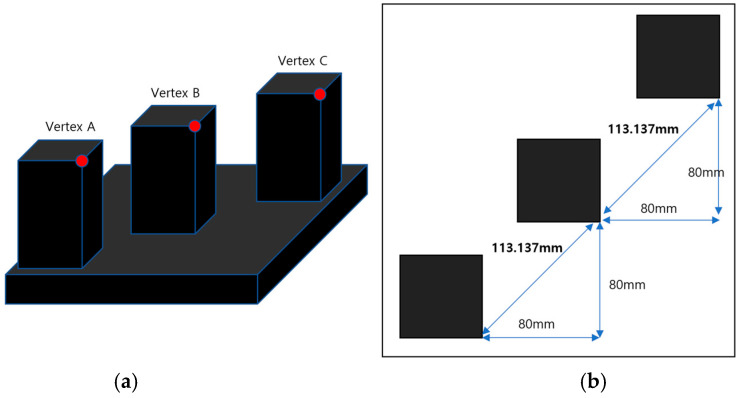
Design of a precisely machined block for measurement accuracy evaluation. (**a**) Design of specimen. (**b**) Distance of each vertex point.

A custom measurement specimen was fabricated to validate the developed target block localization algorithm. The fabrication precision of the specimens was based on the machining accuracy of a precision CNC (Computer Numerical Control) system. The CNC machine used for specimen fabrication provides a machining tolerance of ±0.01 mm. Figure 9 shows the geometries and dimensions of the fabricated specimens. The specimen comprises three aluminum rectangular prisms, each with dimensions of 50 (L) × 50 (W) × 100 (H) mm, rigidly mounted on a single base plate.

The three red points in Figure 9a represent the reference vertices to be extracted using the proposed algorithm. To evaluate the measurement accuracy, the distances between the three vertices, obtained from the point cloud data measured by the 3D laser tracker, were compared with the theoretical distances derived from the machining dimensions of the specimen. As shown in Figure 9b, the theoretical distance between each pair of reference vertices, based on the design and machining specifications, was 113.137 ± 0.01 mm.

### 4.2. Vertex Measurement and Accuracy Evaluation

**Figure 10:**
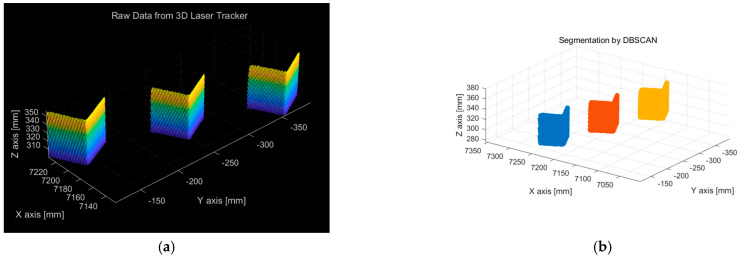
The 3D raw data measured from the manufactured precision-machined block, along with the segmentation results obtained using DBSCAN. (**a**) Raw dataset of 3D point cloud. (**b**) Data segmentation by DBSCAN.

The fabricated test specimen was placed approximately 7.2 m away from the sensor for scanning. The surface of the aluminum material was treated with black anodization. Figure 10a shows the 3D point-cloud data obtained from the scan. The DBSCAN algorithm was applied to distinguish each measured rectangular prism from the raw data. Figure 10b presents the classification results, where the individual measurement regions of the rectangular prisms are separated using the DBSCAN algorithm and visualized in different colors.

**Figure 11:**
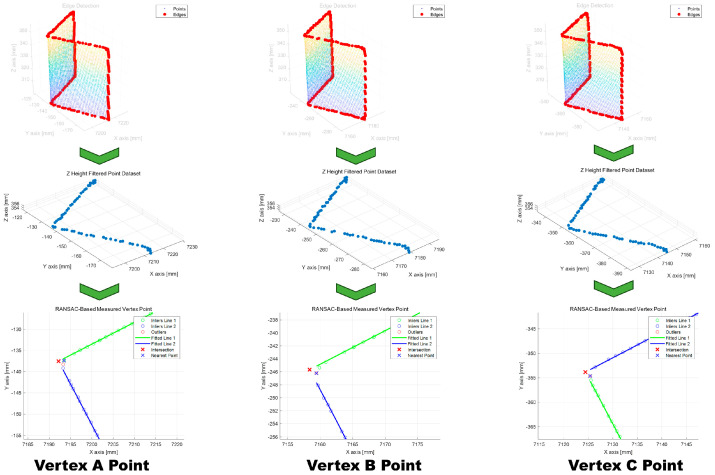
Results of using an algorithm to extract the reference positions from each classified block data.

The previously described algorithm was applied to extract the 3D positions of the vertices from each rectangular prism. First, the edge data were extracted from each segmented point cloud dataset. The extracted edge data were then filtered using a predefined threshold value along the *z*-axis (height). The filtered points were projected onto a 2D plane, and two lines were fitted to the projected data. The intersection point of the two lines was then computed and the point nearest to this intersection was designated as the vertex. Figure 11 shows the results of applying the vertex extraction algorithm to each separate 3D point cloud dataset.

**Figure 12:**
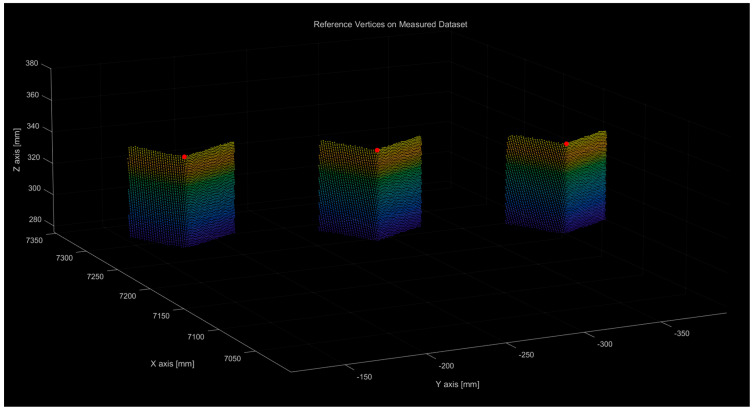
Result of marking each reference location extracted from the raw data of the precisely machined block.

Figure 12 shows the vertex point data representing the reference positions of each rectangular prism overlaid on the measured raw point cloud data. Table 1 lists the measured coordinates of the three vertices. Based on the coordinate information in Table 1, the distances between vertices A, B, and C were calculated using Euclidean geometry.

Table 2 lists the distances between points A and B and between points B and C. The distances measured by the sensor were compared with reference distances derived from the design specifications used to fabricate the test specimens. The difference between the design and measured values is denoted as the error, which is defined as the measurement error of the sensor. A machining tolerance of ±0.01 mm in the fabrication process was considered negligible in the error analysis. The analysis confirmed that the measurement error did not exceed 0.5 mm.

## 5. Conclusions

In this study, a high-precision 3D measurement system and a reference point extraction algorithm are proposed to automate the placement of docking blocks used for large vessels and naval ships. The proposed system is designed and implemented by integrating an algorithm with a mobile robot platform. By mounting a 3D laser tracker sensor onto a mobile robot, the system facilitates flexible adjustment of the measurement positions, thereby enabling high-precision measurements across a wide dry dock area using a single sensor. Owing to the uneven surface quality of conventional docking blocks made of wood or concrete, this study employed precisely machined aluminum target blocks attached to the corners of the docking blocks, which served as representative reference positions. Target blocks were identified from point cloud data acquired by the 3D laser tracker using the DBSCAN clustering algorithm. Subsequently, a corner detection algorithm was developed that automatically extracts edge features and applies RANSAC-based line fitting to identify the intersection vertices and reconstruct their 3D coordinates. To validate the proposed approach, a precision-machined test specimen was fabricated using a CNC system. The measurement results showed that the extracted vertex positions had an error of less than 0.5 mm compared to the theoretical design values, which shows that the proposed system meets the submillimeter precision requirements for docking block placement in large vessel operations.

The proposed mobile robot-based 3D measurement system offers the advantage of high-precision measurements at various locations within a dry dock without relying on multiple fixed sensors. Furthermore, it provides real-time feedback on block placement positions, thereby minimizing installation errors and enhancing operational safety and process efficiency. Future work will focus on verifying the durability and stability of the system through repeated experiments in real dry-dock environments as well as improving its robustness to environmental factors such as lighting variation, dust, and weather conditions. Enhancements to the autonomous navigation accuracy of robots will also be explored.

To conclude, the mobile robot-based high-precision 3D measurement system and reference point extraction algorithm presented in this study offer effective solutions for automating the placement of docking blocks in large vessels and naval ships. The proposed approach is expected to improve the safety and increase the efficiency of the maintenance operations in dry dock.

## Figures and Tables

**Table 1:** The 3D position data extracted by applying the algorithm.

Points	X	Y	Z
Vertex A (mm)	7193.2747	−138.2841	355.2094
Vertex B (mm)	7159.4135	−246.2145	355.1761
Vertex C (mm)	7125.4157	−354.6206	355.1235

**Table 2:** Error values obtained by comparing the measured data with the design values.

Points	A–B	B–C
Reference Distance (mm)	113.1370	113.1370
Measured Value Distance (mm)	113.1174	113.6122
Error Value (mm)	0.0196	0.4752

## Data Availability

Data are contained within the article.
